# Supplementary material for: Content-rich biological network constructed by mining PubMed abstracts
Source: BMC Bioinformatics. 2004 Oct 8;5:147. doi: 10.1186/1471-2105-5-147 (PMC528731; doi:10.1186/1471-2105-5-147)
Supplement: Additional File 2 — The original results of the above study (non-essential files are deleted to keep the file size under the limit set by BMC bioinformatics). [file 1471-2105-5-147-S2.bz2 › chilibotAdditionalFile2/dip40/313ID10606357E1370/html/S100A8_S100A9.html]

 


 **S100A8** and **S100A9** 
  
Found 177 abstracts in PubMed, retrieved 40.  
 

 What does Google say? 
 PDF only 
| .edu only 

---

**Interactive relationship** (e.g. stimulation, inhibition, etc)

**Neutral relationship**- By non radioactive in situ hybridization and immunohistochemistry on serial sections, it was demonstrated that IFNgamma T lymphocytes and  **S100A8** ,  **S100A9**  macrophages embrace granulomas, which consisted of  **S100A8** ,  **S100A9** , HLA DR, CD40, TNFalpha macrophages.  Ref: 12434424 J Pathol, 2002
- MRP 8  [ **S100A8** ]  mRNA but not protein is present in the myeloid cells of these mice, suggesting that the stability of MRP 8  [ **S100A8** ]  protein is dependent on MRP 14  [ **S100A9** ]  expression.  Ref: 12640137 Mol Cell Biol, 2003
- We therefore conclude that the MRP 8  [ **S100A8** ]  14 complex binds to endothelial cells via the MRP 14  [ **S100A9** ]  subunit interacting chiefly with heparan sulfate proteoglycans.  Ref: 11723110 J Biol Chem, 2002
- Moreover, MRP8  [ **S100A8** ]  MRP14  [ **S100A9** ]  initiated oxidase activation on its own, through a calcium dependent specific interaction with cytochrome b558 as shown by Atomic Force Microscopy and a structure function relationship investigation.  Ref: 12719414 J Biol Chem< MedlineTA>J Biol Chem, 2002
- There was also an upregulation of  **S100A8**  A9 or A12 protein, as detected by the antibody MAC387  [ **S100A9** ] , in normal keratinocytes adjacent to both atypical keratinocytes and melanocytes in PAK and LM, respectively.  Ref: 12652189 Am J Dermatopathol, 2003
- Furthermore MRP 8  [ **S100A8** ]  14 and MRP 14  [ **S100A9** ]  did not bind to a glycosaminoglycan minus cell line.  Ref: 11723110 J Biol Chem, 2002
- We subsequently analysed S100A7,  **S100A8** , S100 A9 and S100 A12 in three additional samples and were able to confirm  **S100A8**   **S100A9**  specific over expression in 1q linked pedigrees.  Ref: 12384771 Hum Genet, 2002
- Calgranulins  **S100A8**  and  **S100A9**  are negatively regulated by glucocorticoids in a c Fos dependent manner and overexpressed throughout skin carcinogenesis.The two calgranulins  **S100A8**  and  **S100A9**  were found to be differentially expressed at sites of acute and chronic inflammation.  Ref: 12082614 Oncogene, 2002
- **S100A8** ,  **S100A9**  and the  **S100A8**  A9 heterodimer complex specifically bind to human endothelial cells identification and characterization of ligands for the myeloid related proteins  **S100A9**  and  **S100A8**  A9 on human dermal microvascular endothelial cell line 1 cells.The natural ligands of the S100 EF hand proteins  **S100A8**  and A9 myeloid related proteins 8 and 14 have long been searched for in order to further the understanding of the role of the  **S100A8**  A9 expressing monocyte subpopulation in progressing inflammatory processes.  Ref: 11867565 Int Immunol, 2002
- An abundant 32kDa protein capable of stimulating IL 8 production was isolated from the secretion and identified as neutrophil cytoplasmic protein MRP 14  [ **S100A9** ] , which is the heavy polypeptide chain in the MRP 8  [ **S100A8** ]  14 heterodimer.  Ref: 12748056 Am J Respir Cell Mol Biol< MedlineTA>Am J Respir Cell Mol Biol, 2002
- We demonstrate that  **S100A8** ,  **S100A9**  and the  **S100A8**  A9 heterodimeric complex bind to human dermal microvascular endothelial cell line 1 with an increasing binding capacity progressing from  **S100A8**  <.  Ref: 11867565 Int Immunol, 2002

**Non-interactive relationship** (e.g. studied together, co-existance, homology, etc.)

- It was also possible to detect fibronectin networks and MRP8  [ **S100A8** ] , MRP14  [ **S100A9** ] , and defensin positive granulocytes and macrophages in particular wounds up to 30 days old.  Ref: 12590055 Forensic Sci Int, 2003
- These analyses demonstrated up regulation of  **S100A8** ,  **S100A9**  and, to a lesser extent, S100A7 and S100A12, only in the 1q21 linked family.  Ref: 12384771 Hum Genet, 2002
- The endothelial cell associated ligand proteins isolated by the use of the  **S100A9**  monomer and the  **S100A8**  A9 dimer were subjected to mass spectrometry for protein identification.  Ref: 11867565 Int Immunol, 2002
- Glucocorticoids, which exhibit potent anti inflammatory and anti tumor promoting activities repressed TPA mediated  **S100A8**  and  **S100A9**  induction in wild type, but not in c fos mice, thus identifying both genes as the first examples of AP 1 target genes whose repression of TPA induced transcription by glucocorticoids depends on c Fos.  Ref: 12082614 Oncogene, 2002
- OBJECTIVES To determine whether myeloid related proteins MRP8  [ **S100A8** ]  MRP14  [ **S100A9** ] , a complex of two S100 proteins related to neutrophil and monocyte activation, might be used as a marker for disease activity, and as an early indicator of relapse in juvenile idiopathic arthritis.  Ref: 12594109 Ann Rheum Dis, 2003
- INTERPRETATION Dysregulation of zinc metabolism associated with accumulation in plasma of  **S100A8**  and  **S100A9**  defines a new disease, which encompasses a pathological role for dysregulation of two members of the large S100 protein family.  Ref: 12480428 Lancet, 2002
- A compensatory increase in other proteins was not detected in cells lacking MRP 8  [ **S100A8** ]  and MRP 14  [ **S100A9** ] .  Ref: 12640137 Mol Cell Biol, 2003
- These studies 1 show that  **S100A8** ,  **S100A9** , and  **S100A8**  A9 are potent stimulators of neutrophils and 2 strongly suggest that these proteins are involved in neutrophil migration to inflammatory sites.  Ref: 12626582 J Immunol, 2003
- In a semi recombinant cell free assay, recombinant MRP8  [ **S100A8** ]  MRP14  [ **S100A9** ]  increased the affinity of p67 phox for cytochrome b558 synergistically with p47 phox.  Ref: 12719414 J Biol Chem< MedlineTA>J Biol Chem, 2003
- **S100A8** , the heterodimerization partner of  **S100A9**  was not detectable in peripheral blood cells, suggesting that even a deficiency in both  **S100A8**  and  **S100A9**  proteins was compatible with viable and mature neutrophils.  Ref: 12529407 Mol Cell Biol< MedlineTA>Mol Cell Biol, 2003
- During a transient relapse there was an increase in MRP8  [ **S100A8** ]  MRP14  [ **S100A9** ] .  Ref: 12594109 Ann Rheum Dis, 2003
- Similar results were obtained in the apolipoprotein E knockout mouse model, where preferably recombinant  **S100A9**  but no  **S100A8**  bound to the endothelium of the aorta ascendens.  Ref: 11867565 Int Immunol, 2002
- Expression of MRP8  [ **S100A8** ] , MRP14  [ **S100A9** ] , and MRP8  [ **S100A8** ]  MRP14  [ **S100A9** ]  in synovium was determined by immunohistochemistry.  Ref: 12794836 Arthritis Rheum, 2003
- A novel inflammatory syndrome defined by extraordinarily high expression of  **S100A8**  and  **S100A9**  confirmed recent observations in vitro demonstrating a role of these proteins during recruitment of leukocytes.  Ref: 12697438 Trends Immunol, 2003
- Changing the conformation state of cytochrome b558 initiates NADPH oxidase activation MRP8  [ **S100A8** ]  MRP14  [ **S100A9** ]  regulation.  Ref: 12719414 J Biol Chem< MedlineTA>J Biol Chem, 2003
- **S100A8**  and  **S100A9**  are absent or are expressed at minimal levels in normal epidermis.  Ref: 12704215 J Histochem Cytochem, 2003
- The presence of an active phenotype of macrophages was studied by detection of myeloid related proteins 8 and 14 MRP8  [ **S100A8** ]  and MRP14  [ **S100A9** ] , respectively.  Ref: 12528127 Arthritis Rheum, 2003
- However, detection of MRP8  [ **S100A8** ] , MRP14  [ **S100A9** ]  and defensin provides no advantage over the routine histological detection of granulocytes and macrophages in wounds under 1 2 days old.  Ref: 12590055 Forensic Sci Int, 2003
- These studies also indicate, as reported previously, that S100A7,  **S100A8** , and  **S100A9**  expression is markedly altered in psoriasis, suggesting a role for these proteins in disease pathogenesis.  Ref: 12704215 J Histochem Cytochem, 2003
- In addition, elevated levels of  **S100A9**  and  **S100A8**  in body fluids of inflamed tissues strengthen the view that these molecules are important players in fighting inflammation.  Ref: 12645005 Microsc Res Tech, 2003
- MRP8  [ **S100A8** ]  MRP14  [ **S100A9** ]  purified from neutrophils, enhanced oxidase turnover of B cells in vitro, suggesting that MRP8  [ **S100A8** ]  MRP14  [ **S100A9** ]  is involved in the activation process.  Ref: 12719414 J Biol Chem< MedlineTA>J Biol Chem, 2003
- Here we show a strong up regulation of the RNA and protein levels of the two Ca binding proteins  **S100A8**  and  **S100A9**  in the hyperthickened epidermis of acute murine and human wounds and of human ulcers.  Ref: 11463791 J Biol Chem, 2001
- On the basis of these data, it was hypothesized that in human cat scratch disease,  **S100A8** ,  **S100A9**  macrophages continuously migrate to the granulomas.  Ref: 12434424 J Pathol, 2002
- Concentrations of MRP8  [ **S100A8** ]  MRP14  [ **S100A9** ]  in serum and SF were measured by enzyme linked immunosorbent assay.  Ref: 12794836 Arthritis Rheum, 2003
- HIV 1 transcription and virus production are both accentuated by the proinflammatory myeloid related proteins in human CD4 T lymphocytes. **S100A8** ,  **S100A9** , and S100A12, collectively known as myeloid related proteins, are highly expressed by the myeloid cell lineage and are found in the extracellular milieu during infections and inflammatory conditions.  Ref: 12218151 J Immunol, 2002
- The time dependent expression of fibronectin, MRP8  [ **S100A8** ] , MRP14  [ **S100A9** ]  and defensin in surgically treated human skin wounds.  Ref: 12590055 Forensic Sci Int, 2003
- No differences between the expression of MRP8  [ **S100A8** ]  and MRP14  [ **S100A9** ]  could be demonstrated in the wounds, the majority of which were only several hours old.  Ref: 12590055 Forensic Sci Int, 2003
- RESULTS MRP8  [ **S100A8** ] , MRP14  [ **S100A9** ] , and MRP8  [ **S100A8** ]  MRP14  [ **S100A9** ]  levels were increased in serum, SF, and synovium from PsA, RA, and SpA patients.  Ref: 12794836 Arthritis Rheum, 2003
- In all 3 groups, paired samples of serum and SF showed significantly higher MRP8  [ **S100A8** ]  MRP14  [ **S100A9** ]  levels in SF mean SD 1 10 1 99 ng ml median 1 00 than in serum 908 679 ng ml median 695 P = 0.0001.  Ref: 12794836 Arthritis Rheum, 2003
- Together with  **S100A8**  and  **S100A9** , it belongs to the calgranulin subfamily, i.e. it is mainly expressed in granulocytes, although there is an increasing body of evidence of expression in keratinocytes and psoriatic lesions.  Ref: 12777802 Acta Crystallogr D Biol Crystallogr, 2003
- Furthermore,  **S100A8**  and  **S100A9**  expression was found to be associated with partially differentiated keratinocytes in vitro.  Ref: 11463791 J Biol Chem, 2001
- Analysis by electrophoresis and mass spectrometry showed that the patients protein contained normal  **S100A8**  and  **S100A9**  subunits.  Ref: 12480428 Lancet, 2002
- Granulocytic infiltrates limited to the perivasal space could be detected 20 30 min after infliction of the wound expressing MRP8  [ **S100A8** ] , MRP14  [ **S100A9** ]  and defensin.  Ref: 12590055 Forensic Sci Int, 2003
- Fibronectin, MRP8  [ **S100A8** ] , MRP14  [ **S100A9** ]  and defensin were detected immunohistochemically in 46 surgically treated, fresh hours old human wounds and in wounds of 13 individuals who died immediately from fatal trauma airplane crashes or train rollovers.  Ref: 12590055 Forensic Sci Int, 2003
- By comparing  **S100A8**  and  **S100A9**  mRNA levels in wild type and c Fos deficient mice we found that expression is negatively regulated by c Fos AP 1.  Ref: 12082614 Oncogene, 2002
- Myeloid related protein 14 MRP 14  [ **S100A9** ]  and its heterodimeric partner, MRP 8  [ **S100A8** ] , are cytosolic calcium binding proteins, highly expressed in neutrophils and monocytes.  Ref: 12640137 Mol Cell Biol, 2003
- This factor is composed of 8 and 14 kDa subunits, which have also been termed migration inhibitory factor related proteins MRP8  [ **S100A8** ]  and MRP14  [ **S100A9** ] .  Ref: 12137245 Mediators Inflamm, 2002
- In this study, we report that  **S100A8** ,  **S100A9** , and  **S100A8**  A9 caused neutrophil chemotaxis at concentrations of 10 12 10 9 M.  Ref: 12626582 J Immunol, 2003
- **S100A8**  and  **S100A9**  are small calcium binding proteins that are highly expressed in neutrophil and monocyte cytosol and are found at high levels in the extracellular milieu during inflammatory conditions.  Ref: 12626582 J Immunol, 2003
- Myeloid related proteins MRP8  [ **S100A8** ]  and MRP14  [ **S100A9** ]  of the S100 family are EF hand calcium binding proteins.  Ref: 12719414 J Biol Chem< MedlineTA>J Biol Chem, 2003
- Two S100 proteins, namely  **S100A9**  MRP14  [ **S100A9** ]  and  **S100A8**  MRP8  [ **S100A8** ] , constitute roughly 40% of the cytosolic protein in these cells, implying by their pure abundance an important role in the effector functions of neutrophils.  Ref: 12645005 Microsc Res Tech, 2003
- Microglial cells were visualized by immunolabeling of proteins which are known to be expressed either constitutively or facultatively, such as CD68, major histocompatibility complex class II MHC II, leukocyte common antigen LCA, leukocyte chemotactic factor LCF, macrophage inhibitory factor related protein MRP 8  [ **S100A8** ] , MRP14  [ **S100A9** ] , CD4 and allograft inflammatory factor 1 AIF 1.  Ref: 11307625 Acta Neuropathol Berl , 2001
- During this process, they may be activated by IFNgamma T helper 1 lymphocytes and be differentiated to  **S100A8** ,  **S100A9**  sessile, HLA DR, CD40 antigen presenting, TNFalpha pro inflammatory macrophages forming granulomas.  Ref: 12434424 J Pathol, 2002
- In immediate fatal trauma, it was not possible to detect fibronectin outside of bleeding areas nor could inflammatory cells be visualized in the interstitium using MRP8  [ **S100A8** ] , MRP14  [ **S100A9** ] , or defensin antibodies.  Ref: 12590055 Forensic Sci Int, 2003
- Implication of extracellular zinc exclusion by recombinant human calprotectin MRP8  [ **S100A8** ]  and MRP14  [ **S100A9** ]  from target cells in its apoptosis inducing activity.  Ref: 12137245 Mediators Inflamm, 2002
- Protein complexes formed by  **S100A8**  and  **S100A9**  represent the only AA binding capacity in the human neutrophilic cytosol and are involved in the intracellular arachidonic acid metabolism.  Ref: 12553726 Biol Chem, 2002
- In addition, it was verified by immunohistochemical analysis that the expressions of Calgranulin A  [ **S100A8** ] , Calgranulin B  [ **S100A9** ] , and Cathepsin D were mainly located in cholesteatoma epithelium.  Ref: 12792315 Laryngoscope, 2003
- The brains showed no or a negligible vascular expression of MRP8  [ **S100A8** ]  and MRP14  [ **S100A9** ] .  Ref: 11307625 Acta Neuropathol Berl , 2001
- OBJECTIVE To analyze S 100 protein expression, in the form of myeloid related protein 8 MRP8  [ **S100A8** ] , MRP14  [ **S100A9** ] , and the heterodimer MRP8  [ **S100A8** ]  MRP14  [ **S100A9** ] , in psoriatic arthritis PsA patients compared with rheumatoid arthritis RA and spondylarthropathy SpA patients, and to determine the effect of methotrexate MTX on the MRP antigen expression in PsA patients.  Ref: 12794836 Arthritis Rheum, 2003
- However, neutrophil activation by  **S100A8** ,  **S100A9** , and  **S100A8**  A9 did not induce actin polymerization.  Ref: 12626582 J Immunol, 2003
- **S100A8** ,  **S100A9** , and  **S100A8**  A9 stimulated shedding of L selectin, up regulated and activated Mac 1, and induced neutrophil adhesion to fibrinogen in vitro.  Ref: 12626582 J Immunol, 2003
- ... S100A7,  **S100A8** , and  **S100A9**  are markedly overexpressed.  Ref: 12704215 J Histochem Cytochem, 2003
- Calprotectin  **S100A8**   **S100A9** , an Inflammatory Protein Complex from Neutrophils with a Broad Apoptosis Inducing Activity.  Ref: 12808281 Biol Pharm Bull, 2003
- **S100A9**   **S100A8**  Myeloid representatives of the S100 protein family as prominent players in innate immunity.  Ref: 12645005 Microsc Res Tech, 2003
- CONCLUSIONS MRP8  [ **S100A8** ]  MRP14  [ **S100A9** ]  serum concentration can be used as a marker for disease activity in patients who receive an ASCT for refractory JIA.  Ref: 12594109 Ann Rheum Dis, 2003
- RESULTS The following genes were more highly expressed in arthritis patients than in normal subjects macrophage differentiation marker MNDA, MRP8  [ **S100A8** ]  and MRP14  [ **S100A9** ] .  Ref: 12096225 Rheumatology , 2002
- RESULTS After ASCT, MRP8  [ **S100A8** ]  MRP14  [ **S100A9** ]  serum concentrations in JIA showed a positive correlation with the Child Health Assessment Questionnaire CHAQ.  Ref: 12594109 Ann Rheum Dis, 2003
- **S100A8**  and  **S100A9**  are strongly expressed in the basal and spinous layers in psoriasis involved tissue.  Ref: 12704215 J Histochem Cytochem, 2003
- These results together with previously published data suggest that  **S100A8**  and  **S100A9**  are novel players in wound repair, where they might be involved in the reorganization of the keratin cytoskeleton in the wounded epidermis, in the chemoattraction of inflammatory cells, and or in the defense against microorganisms.  Ref: 11463791 J Biol Chem, 2001
- **S100A8**  and  **S100A9**  did not form these larger complexes.  Ref: 12704215 J Histochem Cytochem, 2003
- The resulting tryptic peptides were sequenced by tandem mass spectrometry, and based on the recovered partial amino acid sequences, three of the tumor specific protein markers were identified as calgranulin A  [ **S100A8** ] , calgranulin B  [ **S100A9** ]  and calgizzarin.  Ref: 11721643 Proteomics, 2001
- Analyses by means of reverse transcription polymerase chain reaction showed enhanced expression of several genes including calgranulin A  [ **S100A8** ] , calgranulin B  [ **S100A9** ] , psoriasin, thymosin beta 10, cathepsin C, cathepsin D, and cathepsin H in cholesteatoma, supporting the findings from the gene array.  Ref: 12792315 Laryngoscope, 2003
- Finally, injection of  **S100A8** ,  **S100A9** , or  **S100A8**  A9 into a murine air pouch model led to rapid, transient accumulation of neutrophils confirming their activities in vivo.  Ref: 12626582 J Immunol, 2003
- Mean MRP8  [ **S100A8** ]  MRP14  [ **S100A9** ]  serum concentrations dropped markedly in the first three months after ASCT p=0.0039 and clinical parameters of disease activity such as CHAQ markedly improved p=0.0039.  Ref: 12594109 Ann Rheum Dis, 2003
- **S100A8**  and  **S100A9**  calcium binding proteins localization within normal and cyclosporin A induced overgrowth gingiva. **S100A8**  and  **S100A9** , also called myeloid related protein 8 and 14, are calcium binding proteins highly expressed in neutrophils, in which they play a key role in the inflammatory progression.  Ref: 12489193 Connect Tissue Res, 2002
- MRP8  [ **S100A8** ]  and MRP14  [ **S100A9** ]  are absent from Epstein Barr virus immortalized B lymphocytes and coincidentally, these cells display weak oxidase activity compared with neutrophils.  Ref: 12719414 J Biol Chem< MedlineTA>J Biol Chem, 2002
- MRP8  [ **S100A8** ]  and MRP14  [ **S100A9** ]  were co isolated from neutrophils on an anti p47 phox matrix with oxidase cytosolic factors and identified by mass spectrometry.  Ref: 12719414 J Biol Chem< MedlineTA>J Biol Chem, 2002
- The occurrence of MAS in three patients in this study was not preceded by significant changes in MRP8  [ **S100A8** ]  MRP14  [ **S100A9** ]  concentration.  Ref: 12594109 Ann Rheum Dis, 2003
- MRP8  [ **S100A8** ]  MRP14  [ **S100A9** ]  levels in serum correlated with systemic parameters of disease activity erythrocyte sedimentation rate ESR r = 0.55, P = 0.005.  Ref: 12794836 Arthritis Rheum, 2003
- Among the genes that were down regulated in FLs were MRP8  [ **S100A8** ]  and MRP14  [ **S100A9** ] , which are involved in adhesion.  Ref: 11756183 Blood, 2002
- METHODS The apoptosis inducing activity of recombinant human MRP8  [ **S100A8** ]  rhMRP8 and recombinant human MRP14  [ **S100A9** ]  rhMRP14 was examined against EL 4 lymphoma cells in vitro.  Ref: 12137245 Mediators Inflamm, 2002
- The Ca2 binding proteins  **S100A8**  and  **S100A9**  are encoded by novel injury regulated genes.To gain insight into the molecular mechanisms underlying cutaneous wound repair, we performed a large scale screen to identify novel injury regulated genes.  Ref: 11463791 J Biol Chem, 2001
- Our present studies compare the level of expression, and cellular localization of S100A7,  **S100A8** ,  **S100A9** , S100A10, and S100A11 in normal and psoriatic epidermis.  Ref: 12704215 J Histochem Cytochem, 2003
- Proinflammatory Activities of S100 Proteins  **S100A8** ,  **S100A9** , and  **S100A8**  A9 Induce Neutrophil Chemotaxis and Adhesion.  Ref: 12626582 J Immunol, 2003
- One reason might be the conformational variability the  **S100A9**  and  **S100A8**  molecules can adopt.  Ref: 12645005 Microsc Res Tech, 2003
- The S100 family heterodimer, MRP 8  [ **S100A8** ]  14, binds with high affinity to heparin and heparan sulfate glycosaminoglycans on endothelial cells.The S100 family proteins MRP 8  [ **S100A8** ]  and MRP 14  [ **S100A9** ]  form a heterodimer that is abundantly expressed in neutrophils, monocytes, and some secretory epithelia.  Ref: 11723110 J Biol Chem, 2002
- The aim of this review is to give an update on the current developments concerning the  **S100A9**   **S100A8**  molecule in biology and medicine.  Ref: 12645005 Microsc Res Tech, 2003
- This was confirmed ex vivo by co transfection of EBV transformed B lymphocytes with genes encoding MRP8  [ **S100A8** ]  and MRP14  [ **S100A9** ] .  Ref: 12719414 J Biol Chem< MedlineTA>J Biol Chem, 2003
- Moreover, MRP8  [ **S100A8** ]  MRP14  [ **S100A9** ]  dimer behaves as a positive mediator of phagocyte NADPH oxidase regulation.  Ref: 12719414 J Biol Chem< MedlineTA>J Biol Chem, 2003
- In contrast, MRP8  [ **S100A8** ] , MRP14  [ **S100A9** ] , and MRP8  [ **S100A8** ]  MRP14  [ **S100A9** ]  expression in the SLL of PsA patients is increased, particularly in perivascular regions, compared with that in RA and SpA patients, suggesting a central role of MRP proteins in transendothelial migration of leukocytes in PsA.  Ref: 12794836 Arthritis Rheum, 2003
- These data identify  **S100A8**  and  **S100A9**  as novel, tumor associated genes and may point to an as yet unrecognized function of both genes in the development of epithelial skin tumors.  Ref: 12082614 Oncogene, 2002
- Together with two other calgranulins,  **S100A8**  and  **S100A9** , it is mostly expressed in human granulocytes, although there is increasing evidence of expression in keratinocytes and psoriatic lesions.  Ref: 12645006 Microsc Res Tech, 2003
- We have now analyzed expression of LCA, CD68, HLA DR, DP, DQ, MRP 8  [ **S100A8** ] , MRP 14  [ **S100A9** ] , LCF and NOS II in macrophages microglial cells in 39 GBM relapses and their matched primary tumors.  Ref: 11859968 J Neurooncol, 2001
- MRP8  [ **S100A8** ]  MRP14  [ **S100A9** ]  serum concentrations were determined by a sandwich enzyme linked immunosorbent assay ELISA as described.  Ref: 12594109 Ann Rheum Dis, 2003
- These included genes involved in cell proliferation and differentiation eg, calgranulin A  [ **S100A8** ] , calgranulin B  [ **S100A9** ] , psoriasin, thymosin beta 10 and cell invasion eg, cathepsin C, cathepsin D, cathepsin H.  Ref: 12792315 Laryngoscope, 2003
- The data indicate that MRP 14  [ **S100A9** ]  and MRP 8  [ **S100A8** ]  are dispensable for many myeloid cell functions.  Ref: 12640137 Mol Cell Biol, 2003
- The increased expression of  **S100A8**  and  **S100A9**  in wound keratinocytes is most likely related to the activated state of the keratinocytes and not secondary to the inflammation of the skin, since we also found up regulation of  **S100A8**  and  **S100A9**  in the epidermis of activin overexpressing mice, which develop a hyperproliferative and abnormally differentiated epidermis in the absence of inflammation.  Ref: 11463791 J Biol Chem, 2001
